# Proenkephalin Derived Peptides Are Involved in the Modulation of Mitochondrial Respiratory Control During Epileptogenesis

**DOI:** 10.3389/fnmol.2018.00351

**Published:** 2018-09-25

**Authors:** Johannes Burtscher, Camilla Bean, Luca Zangrandi, Iwona Kmiec, Alexandra Agostinho, Luca Scorrano, Erich Gnaiger, Christoph Schwarzer

**Affiliations:** ^1^Department of Pharmacology, Medical University of Innsbruck, Innsbruck, Austria; ^2^Department of Biology, University of Padua, Padua, Italy; ^3^Venetian Institute of Molecular Medicine, Padua, Italy; ^4^D. Swarovski Research Laboratory, Department of Visceral, Transplant and Thoracic Surgery, Medical University of Innsbruck, Innsbruck, Austria; ^5^Oroboros Instruments, Innsbruck, Austria

**Keywords:** mitochondria, high-resolution respirometry, enkephalin, delta opioid receptor, granule cell dispersion

## Abstract

Epilepsies are a group of common neurological diseases exerting a strong burden on patients and society, often lacking clear etiology and effective therapeutical strategies. Early intervention during the development of epilepsy (epileptogenesis) is of great medical interest, though hampered by poorly characterized epileptogenetic processes. Using the intrahippocampal kainic acid mouse model of temporal lobe epilepsy, we investigated the functional role of the endogenous opioid enkephalin during epileptogenesis. We addressed three sequential questions: (1) How does enkephalin affect seizure threshold and how is it regulated during epileptogenesis? (2) Does enkephalin influence detrimental effects during epileptogenesis? (3) How is enkephalin linked to mitochondrial function during epileptogenesis?. In contrast to other neuropeptides, the expression of enkephalin is not regulated in a seizure dependent manner. The pattern of regulation, and enkephalin’s proconvulsive effects suggested it as a potential driving force in epileptogenesis. Surprisingly, enkephalin deficiency aggravated progressive granule cell dispersion in kainic acid induced epileptogenesis. Based on reported beneficial effects of enkephalin on mitochondrial function in hypoxic/ischemic states, we hypothesized that enkephalin may be involved in the adaptation of mitochondrial respiration during epileptogenesis. Using high-resolution respirometry, we observed dynamic improvement of hippocampal mitochondrial respiration after kainic acid-injections in wild-type, but not in enkephalin-deficient mice. Thus, wild-type mice displayed higher efficiency in the use of mitochondrial capacity as compared to enkephalin-deficient mice. Our data demonstrate a Janus-headed role of enkephalin in epileptogenesis. In naive mice, enkephalin facilitates seizures, but in subsequent stages it contributes to neuronal survival through improved mitochondrial respiration.

## Introduction

Mesial temporal lobe epilepsy (mTLE) is the most common epilepsy syndrome in adults (Goldberg and Coulter, 2013; Blumcke et al., 2017) and is one of the most refractory forms of human epilepsy (Engel, 2001; Asadi-Pooya et al., 2017). In numerous cases mTLE is an acquired disorder following a severe brain trauma that triggers a cascade of poorly characterized molecular changes (termed epileptogenesis) ultimately leading to the generation of spontaneous recurrent seizures and chronic epilepsy (Pitkänen et al., 2016). Although not yet well understood, chronic stress appears to be a risk factor for the development of epilepsy (Baldin et al., 2017), and individual stressors represent important triggers for seizures (van Campen et al., 2014).

Activation of delta opioid receptors (DOPr) through it’s endogenous ligand enkephalin (Enk) is considered to mediate proconvulsive effect. Loacker et al. (2007) observed a pronounced decrease of seizure threshold after injection of the specific DOPr-agonist SNC80 upon pentylenetetrazole tail-vein infusion in C57BL/6N mice. However, the proconvulsive properties of DOPr-selective agonists appear to be strain and species specific (Clynen et al., 2014). On the other hand, Enk is upregulated in the hippocampus in rats upon stress (Li et al., 2018) and appears to be important for adaptations to stress (Henry et al., 2017, 2018), thus potentially representing a molecular link between the association of stress and seizures/epilepsy.

Furthermore, DOPr activation has been implicated in neuroprotection under conditions of hypoxia (Mayfield and D’Alecy, 1994a,b), ischemia (Gao et al., 2012), and excitotoxicity (Zhang et al., 2000). This effect might be mediated by mitochondrial alterations (Zhu et al., 2011).

Changes of mitochondrial function have been described to occur after seizures and during epileptogenesis. Oxidative stress and mitochondrial dysfunction appear to be important factors in the pathogenesis of epilepsy (Folbergrova and Kunz, 2012; Rahman, 2015). Mitochondria are also intimately involved in pathways leading to neuronal cell death observed in experimental and human epilepsy (Blümcke et al., 1999).

Epileptic patients often exhibit reduced mitochondrial Complex I (CI) activity and defects in CI are potent generators of epileptogenesis (Kunz et al., 2004; Rahman, 2015). In line with this, induction of seizures in rats causes secondary mitochondrial dysfunction, affecting primarily CI (Kudin et al., 2002; Folbergrova et al., 2010).

We investigated the regulation of Enk during epileptogenesis and studied its effect on seizure threshold. Due to our findings, we probed for differential neuropathological and neurochemical outcomes in prepro-Met-Enk-deficient (Enk^-/-^) mice in the kainic acid (KA) model. Hypothesizing Enk to play a role in mitochondrial dysfunction in epilepsy, we compared wild-type (WT) and Enk^-/-^ mice at different time intervals after KA injection applying high-resolution respirometry.

## Materials and Methods

### Animals

Young adult, male C57BL/6 mice (WT) and prepro-Met-enkephalin deficient (Enk^-/-^) mice (König et al., 1996) were used in all experiments. Quantitative real-time PCR (qPCR) applied on cortical and hippocampal samples revealed no differences in opioid receptor mRNA-expression between WT and Enk^-/-^ mice (**Supplementary Figures S2A–F**).

Mice were kept at 23°C with a 12/12 h light/dark cycle and free access to standard laboratory rodent chow and water. All procedures involving animals were approved by the Austrian Animal Experimentation Ethics Board in compliance with the European convention for the protection of vertebrate animals used for experimental and other scientific purposes ETS no.: 123. Every effort was taken to minimize the number of animals used.

### Seizure Threshold and Opioid Receptor Pharmacology

For pentylenetetrazole (PTZ) tail vein infusions, 4–6 mice were used per group. PTZ (10 mg/mL 0.9% saline, pH 7.4) was injected until generalized clonic seizures were displayed. At that point the mice were killed immediately by neck-dislocation. The infused volume of PTZ was used to calculate the seizure threshold (mg PTZ/kg mouse).

The DOPr agonist (+)-4-[(aR)-a-((2S,5R)-4-allyl-2,5-dimethyl-1-piperazinyl)-3-methoxybenzyl]-*N*,*N*-diethylbenzamide (SNC80, dissolved in 1 molar equivalent HCl) and the DOPr antagonist 17-(Cyclopropylmethyl)-6,7-dehydro-4,5α-epoxy-3,14-dihydroxy-6,7-2′,3′-indolomorphinan (Naltrindole, dissolved in water) were purchased from Tocris. Drugs were applied i.p. 30 min before testing (2 mg/kg or 1 mg/kg diluted in saline, respectively).

### Kainic Acid Injections

Mice were sedated with ketamine (160 mg/kg, i.p.; Graeub Veterinary Products, Switzerland) and then deeply anesthetised with sevoflurane (1–3% based on mouse response) through a precise vaporizer (Midmark, United States). All animals received meloxicam (2 mg/kg) 20 min before surgery as analgesic treatment. 50 nL of a 20 mM KA (pH 7.2, Ocean Produce International, Canada) solution was injected in the stratum radiatum of the CA1 region of the left dorsal hippocampus as previously described (Loacker et al., 2007).

### Histology, Cell Counts and Immunohistochemistry

Four–seven animals per interval after KA (2, 7, 14, and 21 days) or saline (21 days, *N* = 3) per genotype were used for histological studies. Animals were killed by an overdose of thiopental (150 mg/kg) and brains were fixed by transcardial perfusion with paraformaldehyde (4% in 50 mM PBS, pH = 7.2). Immunohistochemistry and Nissl staining were performed on 30 μm free-floating coronal sections covering the entire dorsal hippocampus.

For immunohistochemistry the following protocol was applied: After 90 min in blocking solution (10% normal goat serum, 0.3% Triton X-100 in TBS), primary antibodies for neuropeptide Y (1:10000, Immunostar, #22940), prepro-enkephalin (1:2500, ACRIS, #RA14124), Leu-enkephalin (1:1500, ACRIS, #20066), or somatostatin (1:2000, gift by Prof. Sperk, Innsbruck) were applied over night at room temperature, followed by application of horseradish peroxidase conjugated secondary antibodies (1:500, DAKO, #P044801) for 2.5 h at room temperature and 3,3′-diaminobenzidine for detection (Schwarzer et al., 1996). Nissl staining (Paxinos and Watson, 1986) was used to evaluate neuronal numbers and granular cell dispersion.

Cell counts and measurement of granule cell layer area were performed on sections of the dorsal hippocampus covering the range from 1.4 to 2.4 mm caudal to bregma (Paxinos and Franklin, 2001). Mean cell numbers of each brain were taken for stereological and statistical analysis. Cell numbers of non-principal neurons were assessed for CA1, CA3a, CA3b, CA3c and hilus, principal neurons were counted in CA1 over a length of 250 μm, and in CA3a and CA3c over a length of 125 μm covering the whole width of the layer. Granule cell dispersion was measured as the area of the entire granule cell layer from photomicrographs (100× magnification, Zeiss Axiophot 2).

### *In situ* Hybridization

*In situ* hybridizations were performed on frozen-sections (20 μm) obtained from snap-frozen brains in vicinity to the injection site as described elsewhere (Wittmann et al., 2005). 4–7 WT animals per interval were injected with KA (1, 2, 5, 7, 10, 14, and 21 days) or saline (21 days, *N* = 3, time point 0) and used for *in situ* hybridization studies. Single stranded DNA oligonucleotides (5 pmoles) complementary to prepro-dynorphin (5′-GTTCTCCTGGGACCGCGTCACCACCTTGAACTGACGCCGCAG-3′), prepro-neuropeptide Y (5′-GAGGGTCAGTCCACACAGCCCCATTCGCTTGTTACCTAGCAT-3′), and prepro-enkephalin (5′-TCCCTCATCTGCATCCTTCTTCATGAAGCCGCCATACCTCTTGGC-3′) mRNAs were labeled with ^35^S-dATPs (Hartmann Analytic, Germany; 1000 Ci/mmol) using terminal deoxynucleotidyltransferase (Roche, Germany). Hybridization was performed at 52°C for 18 h.

Data analysis was performed with ImageJ (NIH^1^). Relative optical densities (ROD) were calculated from the gray values obtained from autoradiographs of the upper and lower granular cell layer of the dentate gyrus (DG). Means were calculated for values of both layers of the DG, and background obtained over the corpus callosum was subtracted.

### Quantitative Real-Time PCR (qPCR)

Dorsal hippocampi obtained from adult WT and Enk^-/-^ mice (*N* = 4 per condition) were snap-frozen in liquid nitrogen at different time intervals after KA injections and stored at -80°C for quantitative real-time PCR (qPCR). Total RNA was isolated using RNeasy Micro kit (Qiagen) according to the manufacturer’s instructions.

1 μg of total RNA was reverse transcribed to cDNA using the GoScript Reverse Transcription Mix (Promega, Madison, WI, United States) with an Oligo-dT primer. qPCR based on the SYBR Green chemistry (Promega) was carried out using the CFX384 Real-time System (Bio-Rad, Hercules, CA, United States). Of the four biological replicates for each condition three technical replicates were performed. Beta-actin (Actb) was used for normalization of mRNA expression level of the target genes:

Ndufs3 fw 5′-CTGACTTGACGGCAGTGGAT-3′, rv 5′-CATACCAATTGGCCGCGATG-3′; Relnfw 5′-CAAGCCACTGGACCTCACTC-3′, rv 5′-CGCTGTTGCAACTGTCTGTC-3′; Sdhb fw 5′GTCTACCGCTGCCACACC-3′, rv 5′-AGGTCGCCATCATCTTCTTG-3′; Atp6 fw 5′-CCTTCCACAAGGAACTCCAA-3′, Rv 5′-GGTAGCTGTTGGTGGGCTAA-3′; Actb fw 5′-CTGGCTCCTAGCACCATGAAGAT-3′, rv 5′-GGTGGACAGTGAGGCCAGGAT-3′.

Relative quantification was performed using the comparative cycle threshold (*C*t) method after determining the *C*t values for the reference and target gene in each sample, according to the 2^-ΔΔ^*^C^*^t^ method. The expression data were averaged across the technical replicates before comparing between biological replicates.

### High-Resolution Respirometry

Wild-type and Enk^-/-^ mice were sacrificed by neck dislocation (between 9 and 10 am) and the dorsal hippocampi were quickly dissected on ice. Wet tissue was weighed, and the hippocampi were transferred to ice-cold mitochondrial respiration medium MiR06Cr for mechanical permeabilization of the plasma membrane. Respiration was measured at 37°C in the Oroboros O2k (Oroboros Instruments, Austria) in hippocampi of naïve mice (*N* = 4–5 per genotype), 2 or 21 days after KA-injection (5–6 animals per condition and genotype), and 21 days after saline-injection (*N* = 4–5 per genotype), as described previously (Burtscher et al., 2015). An optimized protocol was applied with succinate at a concentration of 50 mM to prevent potential inhibitory effects of 2 mM malate on Complex II. Tissue-mass specific oxygen fluxes were corrected for residual oxygen consumption, *Rox*, measured after inhibition of the mitochondrial electron transfer system, ETS.

For further normalization, fluxes of all respiratory states were divided by ET-capacity to obtain flux control ratios, *FCR*.

Terminology was applied according to http://www.mitoeagle.org/index.php/MitoEAGLE_preprint_2018-02-08 (see also (Lemieux et al., 2017).

The DC^TM^ Protein Assay (Bio-Rad, Hercules, CA, United States) was used for quantification of protein contents of the homogenized samples according to the manufacturer’s protocol.

### Statistical Methods

One-way ANOVA was calculated to compare seizure threshold data. Repeated measures 1-way ANOVA was applied to compare differences in granule cell dispersion across the rostro-caudal axis, regular 2-way ANOVAs were used to compare neuropeptide mRNA-levels during epileptogenesis and for cell dispersion across time-points. Data obtained from *in situ* hybridization, respiration and qPCRs for reelin and ETS components, and cell count data were evaluated by 2-way ANOVA between genotypes and across different time intervals (derived from populations injected separately). *Post hoc* analyses were performed using Tukey’s *post hoc* test.

Repeated measures 2-way ANOVA was performed to assess differences across mitochondrial states between untreated controls of the tested genotypes. Bonferroni corrected *post hoc* test was applied. Data are presented as mean and SEM.

## Results

### Role of Enk in Seizure Threshold and It’s Regulation During Epileptogenesis

To elucidate the role of Enk on seizure threshold and its regulation during epileptogenesis, we applied DOPr pharmacology in combination with pentylenetetrazole-induced seizure threshold and neurochemical methods in the intrahippocampal KA model, respectively.

**FIGURE 1:**
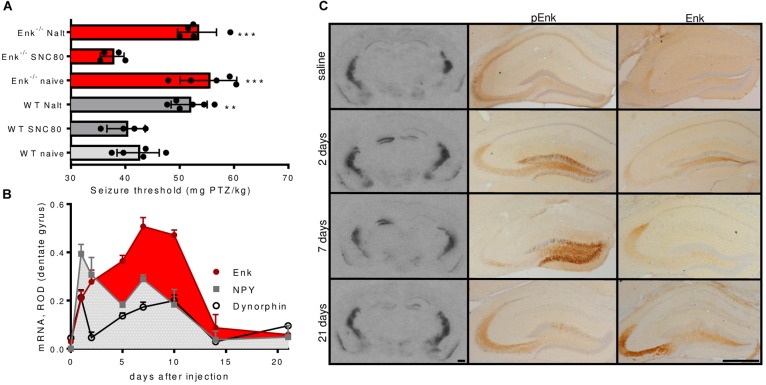
Seizure threshold modulation via the Enk-DOPr system and Enk mRNA- and protein-levels during epileptogenesis. **(A)** Wild-type (WT) mice treated with the DOPr-antagonist naltrindole (Nalt; 1 mg/kg; 30 min before PTZ) exhibited significantly elevated seizure thresholds as compared to untreated WT mice and similar seizure thresholds like untreated Enk-KO mice. By contrast, the DOPr specific agonist SNC80 (2 mg/kg; 30 min before PTZ) did not influence seizure threshold in WT mice but decreased it in Enk-KO mice. Nalt treatment of Enk-KO mice did not further increase seizure threshold. 1-way ANOVA with Tukey’s multiple comparison tests was calculated: *F*(5,23) = 18.54, *P* < 0.001. **(B)** mRNA-regulation of dynorphin, neuropeptide Y (NPY), and enkephalin (Enk) in the dentate gyrus (ROD – relative optical density) during epileptogenesis are depicted. Note the different dynamics of Enk vs. NPY and dynorphin. *Post hoc* significances calculated by two-way ANOVA are omitted for sake of clarity – please refer to main text for statistical information. **(C)** mRNA- levels are shown for representative autoradiographs obtained after *in situ* hybridizations for Met-Enk mRNA close to the injection site of KA (1.8–2.0 mm caudal to bregma, left column). Middle and right columns depict images obtained from KA injected hippocampi after immunohistochemistry for pro-Enk (pEnk) or mature Enk, respectively. Note the marked increase in mRNA and pEnk, but not Enk at early time intervals. Saline-injected controls (3 weeks after injection), 2, 7, and 21 days time intervals after KA-injection are depicted. Scale bars indicate 500 μm. ^∗∗^Indicates a *p*-value < 0.01, ^∗∗∗^*p*-value < 0.001.

The reported proconvulsive actions of Enk are supposed to depend on the activation of DOPr. The threshold for pentylenetetrazole (PTZ)-induced seizures was not altered by pretreatment with the specific DOPr agonist SNC80 (2 mg/kg; 30 min before PTZ) in WT mice. By contrast, pretreatment with the specific DOPr antagonist naltrindole (1 mg/kg; 30 min before PTZ) increased seizure threshold by about 25% (**Figure 1A**). This confirms DOPr mediated proconvulsant effects and suggests that exogenous agonists cannot further enhance the effects of endogenous Enk in WT mice.

Naïve Enk^-/-^ mice displayed a threshold for PTZ-induced seizures comparable to those of WT mice treated with naltrindole (**Figure 1A**). This phenotype was fully reversed upon pre-treatment of Enk^-/-^ mice with SNC80 (2 mg/kg; 30 min pre PTZ). By contrast, pre-treatment with naltrindole (1 mg/kg; 30 min pre PTZ) did not influence the seizure threshold of Enk^-/-^ mice (**Figure 1A**).

Numerous neuropeptides are highly dynamically regulated during status epilepticus and in epileptogenesis, which may impact on the activation of their receptors. Consequently, we investigated Enk mRNA and peptide levels in the unilateral KA model. After KA injection, enkephalin mRNA levels increased continuously, peaking at around 7–10 days after KA-injection in the ipsilateral DG, when first spontaneous seizures occur (Riban et al., 2002). Two-way ANOVA revealed Enk mRNA levels to be significantly higher 5, 7, and 10 days after the initial Enk mRNA increase 1 day after KA-injection. No drop in the silent phase of epileptogenesis, when no or few seizures occur, was apparent. By contrast the mRNA-expression of the endogenous opioid dynorphin and neuropeptide Y (NPY) peaked 1 day after injection and never significantly surpassed that peak thereafter in all time tested intervals. Instead mRNA-expression was significantly decreased 2 and 5 days after KA-injection (**Figure 1B** and **Supplementary Figures S1B–D**). The two-way ANOVA was calculated comparing mRNA-expression of the different neuropeptides across the studied time intervals: *F*_interaction_(14,55) = 7.483, *F*_time-intervals_(7,55) = 27.1, *F*_neuropeptides_(2,55) = 21.5, all *P*s < 0.001. *Post hoc* tests (Tukey’s multiple comparison tests) were applied to compare mRNA-levels across time intervals for individual neuropeptides.

Functionally even more important is the availability of mature, active peptides. Therefore, we compared Enk peptide abundance with alterations observed in mRNA levels in the hippocampus (**Figure 1C** and **Supplementary Figure S1A**). For this purpose, immunohistochemistry, using antibodies specific for pro-Enk (pEnk) and mature Enk was performed at different time intervals of epileptogenesis and assessed qualitatively. Low mRNA levels in saline-injected controls corresponded to low pEnk and Enk peptide levels. After KA injection, pEnk labeling was strongest in ipsilateral granule cell somata after 2 and 7 days. This correlates well, with high mRNA-levels at the respective time intervals (**Figure 1C**). pEnk immunoreactivity was increased in the terminal field of mossy fibers, both ipsi- and contralaterally at most time intervals. However, lowest immunoreactivity was observed in the ipsilateral hippocampus 7 days after KA. Mature Enk immunoreactivity in the mossy fiber terminals in CA3a appeared higher in epileptogenic mice as compared to saline-injected controls. Of note is the fact, that this increase appeared to be only minute at the time intervals of highest mRNA levels (2 days and 7–10 days after injection). This suggests either strong release or mature Enk or inefficient processing of pEnk. Three weeks after KA, Enk mRNA-levels were reduced when compared to earlier time intervals of epileptogenesis. By contrast, pEnk- and Enk-like immunoreactivity was high in mossy fibers (but not somata; **Figure 1C**), suggesting accumulating pro- and mature peptides.

### Effects of Enk-Deficiency in Epileptogenesis

Based on the observed anticonvulsive effects of pharmacological antagonism of DOPr and the prominent continuous up-regulation of Enk mRNA and potentially strong release of Enk early after KA injection, we hypothesized that Enk might be a driving force of epileptogenesis. To address this question, we analyzed neuropathological, morphological and neurochemical alterations in KA injected WT and Enk^-/-^ mice at different time intervals.

**FIGURE 2:**
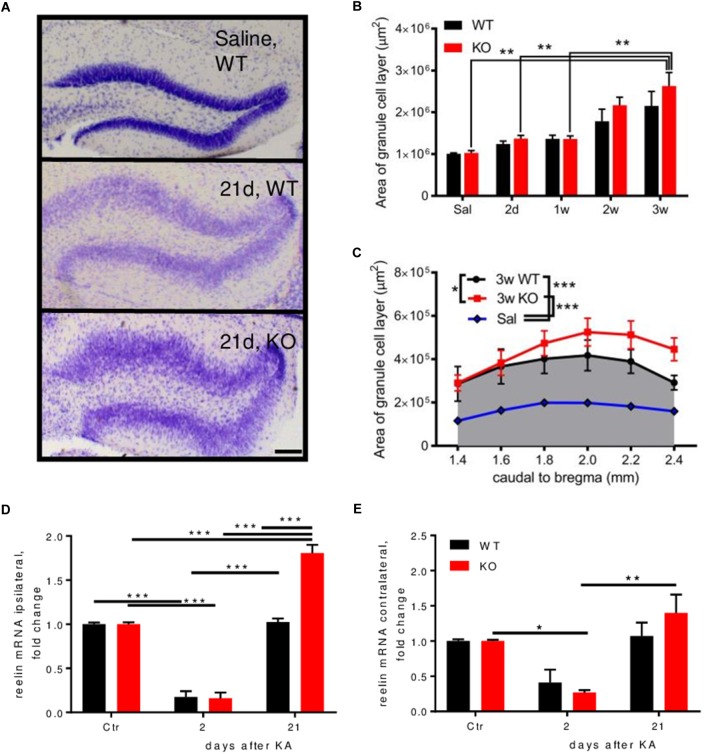
Enk-deficiency aggravates granule cell dispersion during epileptogenesis. **(A)** Granule cell layer dispersion is depicted from Nissl-stained sections of the dentate gyrus of mice 21 days after KA injection. **(B)** Both, WT and KO mice displayed increasing granule cell dispersion (area under the curve across the dorsal hippocampus) after KA-injection. Statistical significance was reached for KO mice; 2 way ANOVA: *F*_interaction_(4,33) = 0.4551, *P* = 0.768, *F*_section_(4,33) = 11.78, *P* < 0.001, *F*_group_(1,33) = 1.80, *P* = 0.189. **(C)** Area covered by the granule cell layer was analyzed from Nissl stained sections. Three weeks after KA injection, granule cells of the dentate gyrus across the dorsal hippocampus were significantly more dispersed in KO mice; 1-way repeated measures ANOVA; *F*_(1.4,7.2)_ = 81.26, *P* < 0.001. Relative expression of reelin mRNA was measured in the ipsi **(D)** and contralateral **(E)** hippocampus. A decrease of reelin mRNA was observed ipsi- **(D)** and for KO contralaterally **(E)** during early epileptogenesis, which was restored to baseline levels in WT mice 3 weeks after KA injection. In KO mice reelin mRNA was significantly overexpressed at this time-interval. 2-way ANOVAs were calculated to assess statistical significances. Interaction, time and group statistics: *F*_(2,12)_ = 30.42, *F*_(2,12)_ = 238, *F*_(1,12)_ = 28.85 (*P* < 0.001 for all) for **(E)** and *F*_(2,12)_ = 1.25 (*P* = 0.321), *F*_(2,12)_ = 18.44 (*P* < 0.001), *F*_(1,12)_ = 0.25 (*P* = 0.627). Scale bar represents 500 μm. ^∗^Indicates a *p*-value < 0.05, ^∗∗^*p*-value < 0.01, ^∗∗∗^*p*-value < 0.001.

Nissl staining was performed to investigate differences between genotypes on cell numbers and morphological alterations during epileptogenesis. Dispersion of the granule cells of the ipsilateral DG occurs 2–3 weeks after KA injection (**Figures 2A,B** and **Supplementary Figure S2G**). When comparing this dispersion between genotypes, we observed an aggravated dispersion in Enk^-/-^ animals across the rostro-caudal axis of the dorsal hippocampus 3 weeks after KA-injection (**Figures 2B,C**). No cell dispersion or differences between genotypes were observed contralaterally. The protein reelin is implicated in granular cell dispersion (Haas et al., 2002). Reelin mRNA was downregulated about fivefold ipsilaterally in both genotypes 2 days after KA (**Figure 2D**). After 21 days, reelin mRNA was on baseline levels in WT, but 1.8-fold overexpressed in Enk^-/-^ mice. Contralaterally (**Figure 2E**), significant alterations of reelin mRNA were observed in Enk^-/-^ mice only.

**FIGURE 3:**
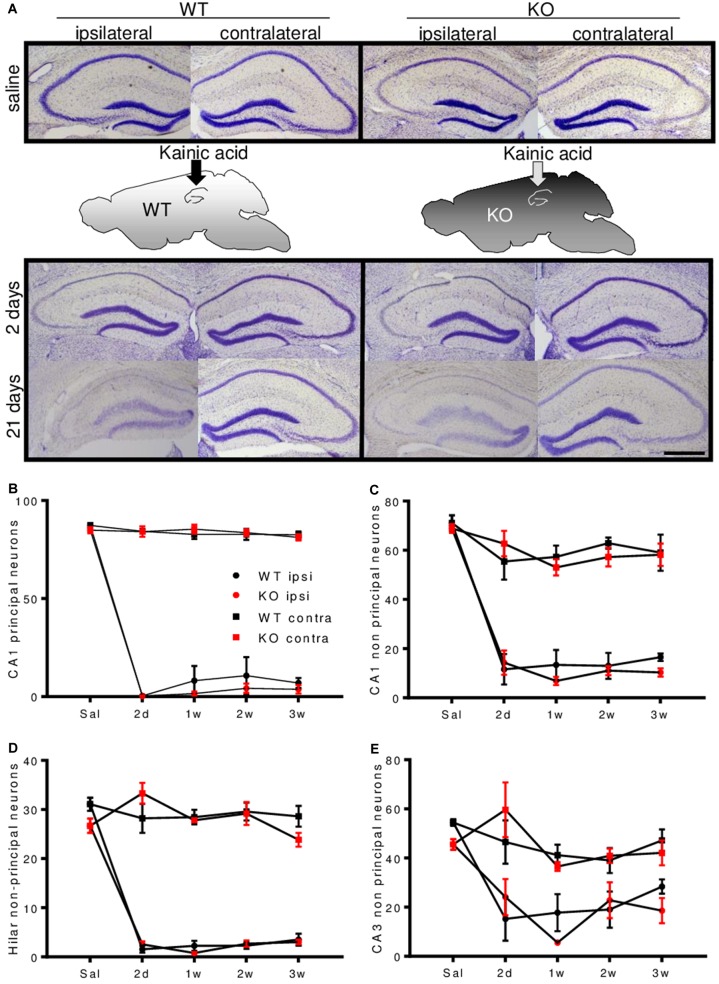
Enkephalin-deficiency does not alter hippocampal cell loss during epileptogenesis. **(A)** Representative Nissl-stained brain sections close to the injection site, after saline or KA-infusion in WT or Enk-/- (KO) mice are depicted. Quantification of cell numbers in hippocampal subfields obtained from Nissl stained sections is shown in **(B–E)**. In all depicted subfields, cell loss was significant and cell numbers remained stable between 2 and 21 days after KA-infusion. Interaction/time/group effects in 2-way ANOVAs were *F*_(12,94)_ = 89.02/*F*_(4,94)_ = 298.6/*F*_(3,94)_ = 984.4 **(B)**, *F*_(12,90)_ = 12.72/*F*_(4,90)_ = 84.54/*F*_(3,90)_ = 147.70 **(C)**, *F*_(12,81)_ = 26.62/*F*_(4,81)_ = 77.38/*F*_(3,81)_ = 342.1 **(D)**, *F*_(12,86)_ = 3.95/*F*_(4,86)_ = 18.97/*F*_(3,86)_ = 29.79 **(E)**, respectively. All *P*-values < 0.001.

Principal neurons in area CA1 and CA3 and non-principal neurons in area CA1 were reduced by 80–100% in the ipsilateral hemisphere. Non-principal neurons in area CA3 were slightly less affected. Cell counts of area CA3 subfields are given in **Supplementary Figures S2H–K**. No marked neuronal loss was observed contralaterally (**Figures 3A–E** and **Supplementary Figures S2G–K**). There were no differences between genotypes.

To investigate, whether highly vulnerable cell types are differentially affected by the lack of Enk, we quantified cell numbers of somatostatinergic interneurons during epileptogenesis. These cells are known to be highly vulnerable in epilepsy (Magloczky and Freund, 1993; Buckmaster et al., 2002). However, we did not observe differences across genotypes (**Supplementary Figure S3**).

### Effects of Enk Deficiency on Mitochondrial Respiration During Epileptogenesis

The lack of beneficial effects paralleled by aggravated morphological alterations in Enk^-/-^ mice suggests that Enk induces not only a reduction in seizure threshold, but also beneficial effects. Reports on the interaction of Enk/DOPr with mitochondrial function and the strong involvement of mitochondrial malfunction in epilepsy stimulated us to investigate this aspect in more detail applying high-resolution respirometry.

**FIGURE 4:**
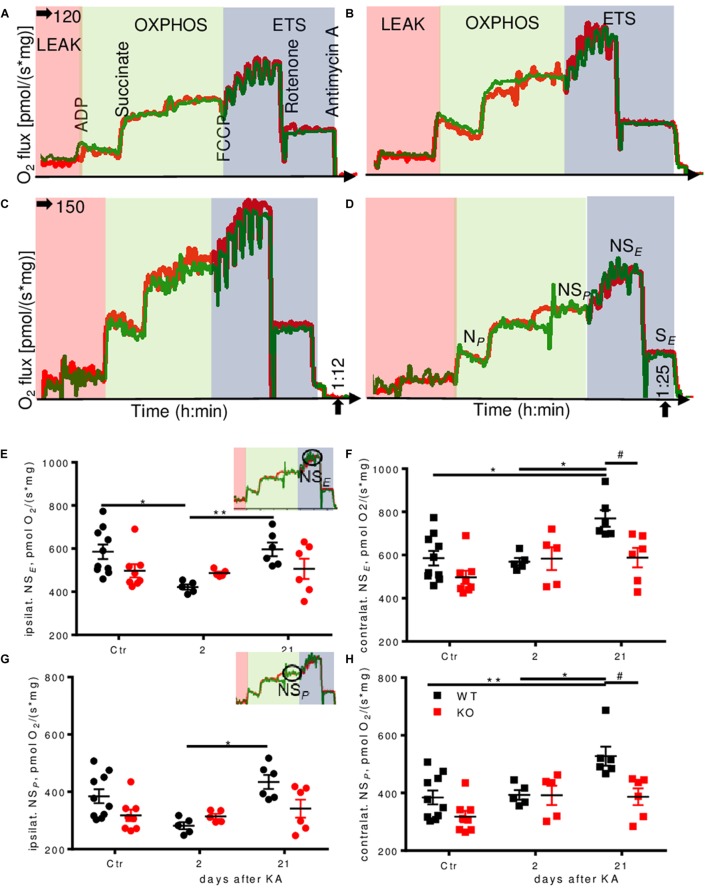
Dynamic regulation of mitochondrial respiration during epileptogenesis in WT, but not Enk-/- (KO) mice. Traces of experimental runs in duplicates (red and green traces) are depicted for WT 2 days **(A)**, KO 2 days **(B)**, WT 21 days **(C)** and KO 21 days **(D)** after KA injection. Data are presented as oxygen flux in pmol/(mg^∗^s), where mg corresponds to wet tissue weight in **(A–D)** and to normalized protein in **(E,F)**. Electron transfer capacity (NS*_E_*) **(E,F)** and OXPHOS capacity (NS*_P_*) **(E,F)** per mg protein ipsi- (**E,G**, respectively) and contralaterally (**F,H**, respectively) are shown with respect to the site of KA-injection. Interaction/time/genotype statistics were as follows: *F*_(2,34)_ = 2.98, *P* = 0.064/*F*_(2,34)_ = 4.24, *P* = 0.023/*F*_(1,34)_ = 1.84, *P* = 0.184 for **(E)**, *F*_(2,34)_ = 2.77, *P* = 0.077/*F*_(2,34)_ = 7.33, *P* = 0.002/*F*_(1,34)_ = 7.20, *P* = 0.011 for **(F)**, *F*_(2,34)_ = 3.19, *P* = 0.054/*F*_(2,34)_ = 5.92, *P* = 0.006/*F*_(1,34)_ = 4.48, *P* = 0.042 for **(G)**, and *F*_(2,34)_ = 2.84, *P* = 0.073/*F*_(2,34)_ = 8.52, *P* = 0.001/*F*_(1,34)_ = 9.62, *P* = 0.004 for **(H)**. ^∗^ and ^#^ indicate a *p*-value < 0.05, ^∗∗^*p*-value < 0.01.

Untreated Enk^-/-^ mice displayed lower respiration as compared to untreated WT mice in some respiratory states, in particular for the electron transfer (ET)-capacity with NADH-linked substrates and succinate combined (NS*_E_*) (10% reduction; **Supplementary Figures S4G,K,L**). Pronounced differences were also observed during epileptogenesis (representative respirometry traces depicted in **Figures 4A–D**). Two days after KA injection, we observed >25% decrease in ET-capacity per mg protein in ipsilateral hippocampi of WT animals (**Figure 4E**) that was restored to baseline levels 3 weeks after injection. Contralaterally (**Figure 4F**), ET-capacity was unchanged 2 days after injection, but increased by 25% 3 weeks after injection. No such effects were observed in Enk^-/-^ mice.

Alterations in oxidative phosphorylation (OXPHOS) capacity (NS*_P_*) per mg protein (**Figures 4G,H**) followed a very similar pattern as ET-capacity.

**FIGURE 5:**
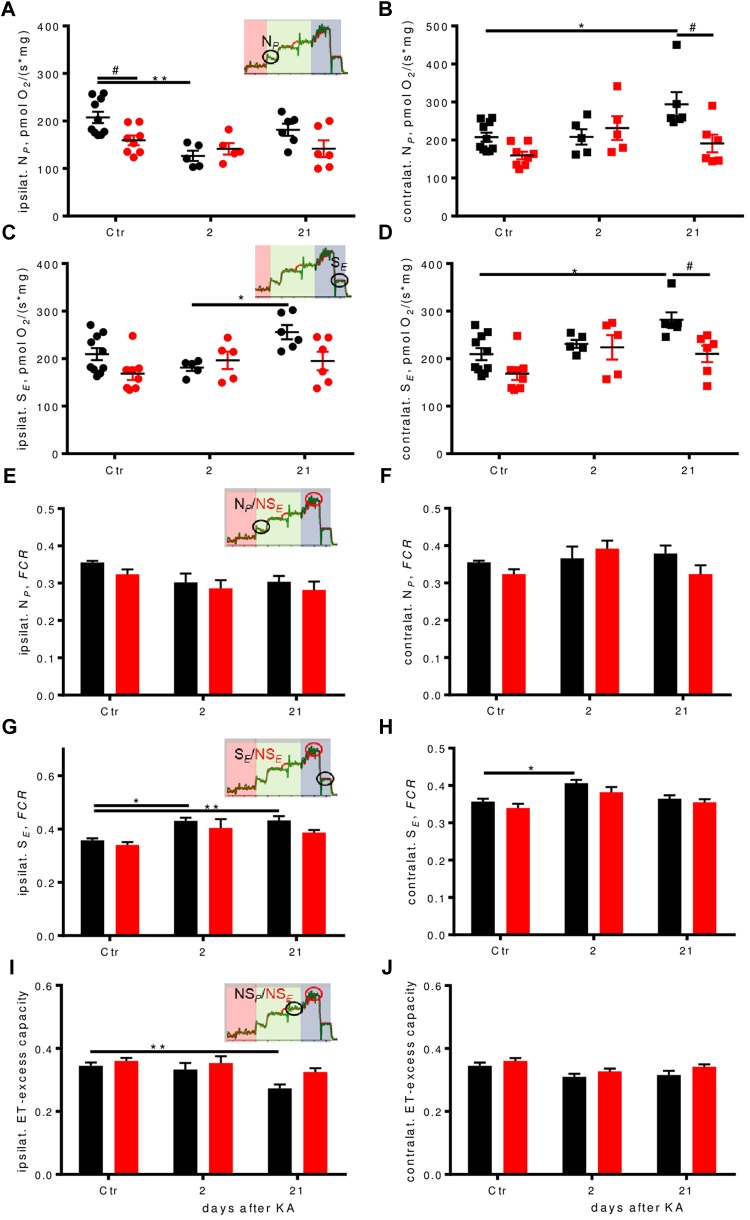
Respiration pathways are differentially affected during epileptogenesis. **(A–D)** Ipsilateral absolute depression in early epileptogenesis and contralateral absolute respirational increase in WT is absent in Enk^-/-^ (KO) mice. N_P_-**(A,B)**, and S_E_-**(C,D)** respiration per mg protein of ipsilateral and contralateral dorsal hippocampi. Interaction/time/genotype statistics: *F*_(2,34)_ = 3.08, *P* = 0.059/*F*_(2,34)_ = 7.11, *P* = 0.003, *F*_(1,34)_ = 5.03, *P* = 0.032 for **(A)**, *F*_(2,34)_ = 3.99, *P* = 0.028/*F*_(2,34)_ = 4.79, *P* = 0.015/*F*_(1,34)_ = 6.25, *P* = 0.017 for **(B)**, *F*_(2,34)_ = 2.87, *P* = 0.070/*F*_(2,34)_ = 3.81, *P* = 0.032/*F*_(1,34)_ = 5.34, *P* = 0.027 for **(C)**, and *F*_(2,34)_ = 1.73, *P* = 0.193/*F*_(2,34)_ = 7.67, *P* = 0.002/*F*_(1,34)_ = 9.18, *P* = 0.005 for **(D)**. **(E–J)** Flux control ratios (FCRs) in WT, but not in Enk^-/-^ (KO) mice change across epileptogenesis. N_P_ FCR **(E,F)** remains similar across epileptogenesis and genotypes, S_E_ FCR **(G,H)** is increased in epileptogenesis in WT. Apparent electron transfer-excess capacity decreases in WT in late epileptogenesis ipsilaterally **(I)**, but not contralaterally **(J)** or in KO. Interaction/time/genotype statistics: *F*_(2,34)_ = 0.124, *P* = 0.883/*F*_(2,34)_ = 6.41, *P* = 0.004/*F*_(1,34)_ = 3.03, *P* = 0.091 for **(E)**, *F*_(2,34)_ = 2.18, *P* = 0.129/*F*_(2,34)_ = 2.35, *P* = 0.110/*F*_(1,34)_ = 1.76, *P* = 0.193 **(F)**, *F*_(2,34)_ = 0.50, *P* = 0.610/*F*_(2,34)_ = 14.50, *P* < 0.001/*F*_(1,34)_ = 5.73, *P* = 0.022 for **(G)**, *F*_(2,34)_ = 0.21, *P* = 0.815/*F*_(2,34)_ = 10.34, *P* < 0.001/*F*_(1,34)_ = 4.17, *P* = 0.049 for **(H)**, *F*_(2,34)_ = 1.05, *P* = 0.360/*F*_(2,34)_ = 8.69, *P* < 0.001/*F*_(1,34)_ = 6.47, *P* = 0.016 for **(I)**, and *F*_(2,34)_ = 0.142, *P* = 0.868/*F*_(2,34)_ = 5.60, *P* = 0.008/*F*_(1,34)_ = 4.74, *P* = 0.037 for **(J)**. ^∗^ and ^#^ indicate a *p*-value < 0.05, ^∗∗^*p*-value < 0.01.

NADH-pathway (N) capacity was significantly higher (by about 30%) in WT controls than in Enk^-/-^ ipsilaterally, but dropped to similar levels 2 days after KA injection, before it recovered almost to baseline level 21 days after injection (**Figure 5A**). Contralaterally, an up-regulation of N-respiration by about 40% was apparent in WT 21 days after injection (**Figure 5B**). No such dynamics were observed in Enk^-/-^ mice.

Succinate-pathway (S) capacity was increased 21 days after injection in WT both ipsi- (**Figure 5C**) and contralaterally (**Figure 5D**) by about 20 and >30%, respectively. Statistically significant higher respiration as compared to Enk^-/-^ was observed only contralaterally.

In line with the reduced respiratory activity in Enk^-/-^ mice (**Supplementary Figure S4G**), overall Complex IV (CIV) activity appeared lower in Enk^-/-^ (**Supplementary Figures S4I,J**), without reaching *post hoc* significance for individual time-points.

The higher dynamic regulation of the OXPHOS system in WT animals matched the mRNA-expression of selected subunits of respiratory Complexes I (NDUFs3), CII (SDHb), and F-ATPase (ATP6), when normalized to beta-actin, in particular for NDUFs3 contralaterally (**Supplementary Figures S4A–F**): NDUFs3 (CI) mRNA was upregulated around fivefold, SDHb (CII) three–fourfold, and ATP6 (ATP-synthase) four–sixfold, both in the hippocampus of KA-injection and the contralateral hippocampus 2 days post injection in WT. This correlated well with especially contralateral respiration upregulation for CI (**Figure 5B**) and CII (**Figure 5D**). Except for ATP6 (which was also upregulated in KO about four–fivefold), the tested respiratory complex subunits in Enk^-/-^ mice were upregulated to smaller extend (about two–threefold) 2 days post injection. Transcription dropped to about one–twofold in both phenotypes 3 weeks after KA for NDUFs4 and SDHb. ATP6 remained elevated (two–threefold) in KO animals.

### Qualitative Changes in the Regulation of Oxidative Phosphorylation in Enk^-/-^ Mice

In order to assess qualitative differences in oxidative phosphorylation between the genotypes, respiration was expressed as *FCR*s by normalization to the respective ET-capacities. In KA treated animals, we observed a significant drop in N-linked *FCR*s ipsilaterally (**Figure 5E**, no *post hoc* significances) and no differences contralaterally (**Figure 5F**). S-respiration *FCRs* were increased in WT mice ipsilaterally, with strong time effects (**Figure 5G**). Enk^-/-^ mice S-respiration *FCRs* displayed a similar tendency trend without reaching statistical significance. Besides time also effects of genotype were significant contralaterally (**Figure 5H**), with Enk^-/-^ mice exhibiting lower S-linked *FCR*. The increase of *FCR* 2 days after KA was significant for WT mice only. The apparent ET-excess capacity (NS*_P_*/NS*_E_*) was reduced by 20% 3 weeks after injection ipsilaterally (**Figure 5I**), in WT, but not Enk^-/-^ mice. No differences in apparent ET-excess capacity were observed contralaterally (**Figure 5J**).

## Discussion

Based on observations of involvement of pharmacological modulation of DOPr in seizure threshold, we aimed to elucidate the role of DOPr’s preferential endogenous ligand, Enk, in epileptogenesis. We report a dual role of the DOPr/Enk system; proconvulsive properties on one hand, enhancement of mitochondrial function on the other.

### DOPr/Enk System Effects on Seizure Threshold and Its Regulation During Epileptogenesis

Pharmacological inhibition of DOPr (**Figure 1A**) such as genetic ablation of Enk (**Figure 2A**) significantly increased seizure threshold, confirming proconvulsive properties of DOPr activation. We therefore hypothesized, that Enk might be involved in the development of epilepsy. To this end we applied the KA model of epileptogenesis and first studied the regulation of Met-Enk transcription in the hippocampus after unilateral KA infusion. Alterations of Enk-contents in the hippocampus after motor-seizures have already been reported in rats (Hong et al., 1980). Indeed, we observed a particular, continuous upregulation of Met-Enk mRNA, exhibiting different dynamics than other neuropeptides, such as neuropeptide Y and dynorphin (**Figure 1B**). Both of these neuropeptides are regulated during epileptogenesis in a seizures-dependent manner. By contrast, Met-Enk mRNA was upregulated continuously during the 1st days after KA infusion, which granted us to suppose, it might act as a driving force in epileptogenesis.

Mature Enk immunoreactivity correlated weakly with the time-course of pEnk mRNA regulation. This might be due to a strong release of Enk or impaired processing resulting in low mature Enk-like immunoreactivity as compared to mRNA and pEnk levels. Fast turnover rates of Enk (Hughes et al., 1975; Simantov and Snyder, 1976) and low pEnk immunoreactivity in the terminal field of mossy fibres as compared to hilus observed 7 days after KA support the first suggestion.

Of note is the fact, that C57BL/6J mice used in this study differ markedly from C57BL/6N mice used in our previous study (Loacker et al., 2007). C57BL/6J mice displayed an increased seizure threshold upon treatment with the DOPr antagonist naltrindole, while C57BL/6N mice did not. By contrast, C57BL/6J mice displayed unchanged seizure threshold upon treatment with the DOPr agonist SNC80, while C57BL/6N mice showed a decrease in seizure threshold. This is on one hand surprising, on the other is in line with reports on strain-dependent differences in the effect of DOPr activation on seizure (Clynen et al., 2014). It is tempting to speculate, that endogenous Enk is sufficient to fully activate DOPr in C57BL/6J, but not in C57BL/6N mice.

### Is Enkephalin a Driving Force in Epileptogenesis?

Both, its apparent proconvulsive effect, and its continuous up-regulation during epileptogenesis suggest Enk as one driving force for those ill-defined molecular events transforming a non-epileptic brain into an epileptic one. Therefore, we applied the KA-model to Enk-deficient mice, expecting to observe mitigated neuropathology (including reduced cell loss) due to reduced seizure frequency and severity.

Surprisingly, there were no differences in cell loss between wild-type and Enk^-/-^ mice at different stages of epileptogenesis. Analyses of epileptogenesis-related neuropathological alterations revealed, however, an increased extent of granule cell dispersion in Enk^-/-^ mice. Enk-deficiency unexpectedly appeared to not only fail to halt cell loss after KA-infusion, it even exacerbated this patho-morphological symptom. As granule cell dispersion has been linked to reductions in reelin expression (Haas et al., 2002), we investigated, whether this was also the case here. Although we did not observe differences in hippocampal reelin immunoreactivity between naive WT and Enk^-/-^ mice, a clear decline of reelin mRNA levels in early intervals was apparent. This is in line with the results described by Heinrich et al. (2006). Reelin mRNA was restored to basal levels in WT mice, but significantly increased in Enk^-/-^ mice 3 weeks after KA infusion (**Figures 2D,E**). Increased granule cell dispersion associated with overexpression of reelin mRNA, but unchanged reelin immunoreactivity points to a functional deficit. However, clarifying the underlying mechanisms is beyond the scope of this study.

### Enkephalin’s Link to Mitochondrial Function During Epileptogenesis

Instead we were curious, how Enk confers protective effects on hippocampal morphological integrity and reelin regulation. A prominent role of mitochondrial dysfunction in temporal lobe epilepsy is well accepted [for reviews see (Kudin et al., 2002; Rowley and Patel, 2013)]. Based on reported links of the Enk/DOPr system with mitochondrial function (Zhu et al., 2009, 2011), we suspected mitochondrial parameters to be involved.

Respiratory deficiencies (Baron et al., 2007), metabolic changes (Giménez-Cassina et al., 2012) and morphological alterations of mitochondria (Gao et al., 2014) have all been implicated in epilepsy. DOPr-mediated neuroprotection has been described for various conditions relevant for epileptic seizures (such as hypoxia, ischemia, excitotoxicity, and oxidative stress), and might be mediated via mitochondrial function. For example, positive effects of hypoxic preconditioning depend on DOPr (Mayfield and D’Alecy, 1994b), Gao et al. (2012) described the adaptation of the DOPr-system by hypoxic preconditioning, which decreases ischemic injury in the hippocampus and Zhang et al. (2000) showed that DOPr-activation, but not activation of other opioid receptors, is protective in excitotoxic injury. Zhu et al. (2011) suggested that DOPr-activation positively affects acute mitochondrial dysfunction through stabilization of mitochondrial membrane potential and calcium levels. Both mechanisms could ultimately play pivotal roles in the maintenance of OXPHOS capacity. As a side note, overexpression of pre-pro-Enk in the striatum of a Huntington’s disease mice model has been shown to improve behavioral deficits and might exert neuroprotective effects (Bissonnette et al., 2013).

For these reasons, we investigated mitochondrial function in naïve mice, at early (2 days after KA) and late (3 weeks after KA) time intervals and compared effects between Enk^-/-^- and WT-mice. We applied functional respirometry applying a substrate-uncoupler-inhibitor-titration (SUIT) protocol to study different respiratory states. Mitochondrial respiration was significantly lower in dorsal hippocampi of naïve Enk^-/-^- than of WT-mice (**Supplementary Figure S4G**). This was not due to specific mitochondrial complex deficiencies since *FCR*s (independent of density effects, due to internal normalization for ET-capacity) were similar between genotypes, including the apparent ET-excess capacity over the phosphorylation system (NS*_P_*/NS*_E_*) (**Supplementary Figure S4H**).

In WT mice, we observed a pronounced reduction of the ET-capacity 2 days after KA-injection, which returned to control-levels 3 weeks after KA ipsilaterally. The patterns were similar for most of the other respiratory states (N-, S-, and NS-respiration, but not for CIV activity).

These dynamic changes in respiration were not observed in Enk^-/-^-animals. Three weeks after KA-injection mitochondrial respiration contralaterally were significantly higher in WT-mice (about 30–50%) than in KO-mice (N*_P_*-, S*_E_*-, NS*_P_*-, and NS*_E_*-states).

The ratio of respiration of mitochondria in the coupled versus uncoupled state is an indicator of the apparent ET-excess capacity, which was significantly reduced in WT- but not in KO-mice ipsilaterally 3 weeks after KA (**Figure 5I**). This suggests alterations that made WT-mice use OXPHOS more effectively.

Such adaptations might be due to enhanced availability or activity of components of the mitochondrial phosphorylation system. Therefore, we applied qPCRs to investigate potential upregulation of gene transcription of selected components of the phosphorylation system. The dynamic mitochondrial respiratory control patterns indeed correlated with a stronger upregulation of these mRNAs in WT mice, supporting the notion of enhanced respiratory capacity. Interestingly, ATP6 mRNA remained high in KO, both ipsi- (**Supplementary Figure S4C**) and contralaterally (**Supplementary Figure S4F**), potentially as a mechanism to compensate for the lack of the adaptation of respiratory excess capacity seen in WT mice (**Figure 5I**).

The upregulation of OXPHOS in the contralateral hippocampus of WT mice 3 weeks after KA was preceded by increased mRNA levels of Ndufs3 and Sdhb 2 days after KA (**Supplementary Figures S4D,E**). It might appear surprising that the contralateral hippocampus seemed to be affected stronger in WT mice during epileptogenesis regarding OXPHOS-parameters (in contrast to neuropeptide regulation). On one hand, we attribute the fewer statistically significant effects ipsilaterally to potentially stronger phasic molecular events triggered by status epilepticus and the higher variability ipsilaterally in terms of seizure activity and cell loss typical for this model of TLE. On the other hand, hemispheric cross-talk plays an important role in preconditioning, possibly linked to Enk and mediated through hemodynamic mechanisms (Iordanova et al., 2018) induced by seizure-activity (Osharina et al., 2017). Cross-hemispheric synchronization has been demonstrated via hemodynamics and arteriole diameter (Mateo et al., 2017); it is no surprise that blood-flow dependent OXPHOS-related genes presumably respond strongly to changes in hemodynamics.

In other disease models cross-hemispheric adaptations to stressors have been described in more detail; Weilnau et al. (2018) for example report cross-hemispheric preconditioning effects following unilateral intrastriatal infusion of 6-hydroxy-dopamine in mice via upregulation of preconditioning-relevant proteins. Contra-lateral insult-related changes in metabolite levels were reported by Ruan et al. (2017) in a stroke model in rats. Our findings support and expand these results by demonstrating contralateral upregulation of OXPHOS mRNA-levels and function in the intrahippocampal KA model of unilateral insult.

## Conclusion

Taken together, our data suggest that the functional role of the Enk/DOPr system in epileptogenesis is more complex than considered. Despite proconvulsive function, the system might be involved in mitigating neuropathology. Here we report the particular regulation of Enk during epileptogenesis and its role in dynamic mitochondrial alterations. More research is required to confirm the suggested link to DOPr-related conditioning effects. Together with recent reports on the role of enkephalin in stress adaptation (Henry et al., 2017, 2018) and the findings, that stress enhanced neuropathological alterations in the hippocampus in epileptogenesis (van Campen et al., 2018), the importance of understanding the enkephalin/DOPr system in epileptogenesis and epilepsy becomes evident. Even more, as there is an apparent link between stress and mitochondrial function (Henningsen et al., 2012). The Enk/DOPr system and its pivotal role in adaptations to one of the most important triggers of seizures and potential risk factor for epilepsies, stress, and its prominent effects on energy metabolism represents an attractive novel molecular target to reduce seizure-induced neuronal damage and potentially disease-modifying therapy.

## Author Contributions

JB performed most of the experimental work and contributed to the writing of the manuscript. CB performed qPCR and related data analysis for mitochondrial mRNAs. LZ, IK, and AA performed some of the surgeries and parts of histochemical analysis. LS supervised the qPCR study. EG designed, supervised, and discussed the respirometry experiments. CS designed and supervised the project, performed the PTZ experiments, and contributed to the manuscript.

## Conflict of Interest Statement

EG is CEO of Oroboros Instruments. The remaining authors declare that the research was conducted in the absence of any commercial or financial relationships that could be construed as a potential conflict of interest.

## Footnotes

1 http://rsb.info.nih.gov/ij

## Supplementary Material

FIGURE S1 (Related to **Figures 1–3**). Enkephalin (Enk) protein- and mRNA-levels during epileptogenesis are depicted. Images in **(A)** represent contralateral hippocampi stained for pro-Enk (pEnk) or mature Enk (Enk) after saline or kainic acid (KA) injection at different time intervals. mRNA-levels in the dentate gyrus of WT mice for dynorphin **(B)**, neuropeptide Y (NPY, **C**), and Enk **(D)** during epileptogenesis are depicted as relative optical densities measured from film autoradiographs obtained by radioactive *in situ* hybridization. Scale bar in **(A)** indicates 500 μm.

FIGURE S2 (Related to **Figure 2**). mRNA-levels of opioid receptors in WT and Enk^-/-^ mice (KO) and additional data on cell numbers are depicted. mRNA levels were assessed for delta-, mu-, and kappa-opioid receptors (DOPr, MOPr, KOPr, respectively) in the hippocampus and cortex of young adult male mice **(A–F)**. Nissl-stained hippocampal sections 1 week (1w) and 2 weeks (2w) after KA injection **(G)** show the onset of granule cell dispersion. Cell counts of area CA3 subfields are given in **(H–K)**. Scale bar indicates 500 μm. **(A–F)** Cortex and hippocampi from naive adult WT and Enk^-/-^ mice (*N* = 4) were analyzed for expression of mu, delta, and kappa opioid receptors (Taqman primer sets Mm01188089_m1; Mm01180757_m1; Mm01230885_m1; respectively; Thermo Fisher) by qPCR. Reactions were performed in a MicroAmp Fast Optical 96-Well Reaction Plate (Applied Biosystems) using the 7500 Fast Real-Time PCR System (Applied Biosystems). Samples were run in duplicates using 50 ng of total RNA equivalents (cDNA). Positive and negative controls were included in all experiments. Threshold cycle (CT) values were recorded as a measure of initial template concentration. Relative levels of RNA were calculated by the ΔΔ*C*_T_ method using SDHa as a reference standard gene. The fold-difference expression was calculated relative to a calibrator sample by 2^-ΔΔ^*^C^*^T^.

FIGURE S3 (Related to **Figure 3**). Numbers of somatostatin immuno-positive neurons were analyzed from WT and Enk^-/-^ mice (KO) in the hippocampal subfields at different time-intervals after KA. Almost complete cell loss was observed ipsilaterally **(A,C,E)**. Somatostatin positive neurons were mostly conserved contralaterally **(B,D,F)**. No genotype specific differences in cell numbers were detected applying 2way ANOVA on distinct hippocampal areas. *N* per condition = 3–4. Ipsilaterally (left panels), time effects were significant in all hippocampal areas (*F* = 157.1; *F* = 17.6; *F* = 119 from top to bottom, *P* in all cases < 0.0001), but not genotype- or interaction-effects. Cell numbers represent area under the curve (AUC) across six hippocampal sections from 1.4 to 2.4 mm caudal to bregma.

FIGURE S4 (Related to **Figures 4**, **5**). mRNA levels of selected subunits of respiratory complexes of WT and enkephalin deficient mice (KO), normalized to beta-actin and basal levels of untreated animals are depicted: NDUFs3 **(A,D)**, SDHb **(B,E)**, and ATP6 **(C,F)**. Data from **(A–C)** have been obtained from the hippocampus of the hemisphere of injection, data from **(D–F)** from the contralateral hippocampus. Absolute oxygen fluxes **(G)** of naïve WT and KO mice differed in a 2way repeated measures ANOVA [*F*_interaction_(4,64) = 3.51, *P* = 0.012/*F*_state_(4,64) = 1073, *P* < 0.001, *F*_genotype_(1,16) = 7.52, *P* = 0.015], but not flux control ratios (FCRs, **H**). No changes were observed for Complex IV respiration across genotypes and time intervals after KA **(I,J)**. Representative traces of respiration protocols for naïve controls are depicted for WT **(K)** and KO **(L)**. NADH-linked respiration (N*_L_* and N*_P_*), OXPHOS capacity (NS*_P_*), ET capacity (NS*_E_*), succinate-linked respiration (S*_E_*). ^#^*P* < 0.05, ^∗^*P* < 0.05, ^∗∗^*P* < 0.01, and ^∗∗∗^*P* < 0.001.
