# Supplementary figures and images for: Proenkephalin Derived Peptides Are Involved in the Modulation of Mitochondrial Respiratory Control During Epileptogenesis
Source: Front Mol Neurosci. 2018 Sep 25;11:351. doi: 10.3389/fnmol.2018.00351 (PMC6167428; doi:10.3389/fnmol.2018.00351)

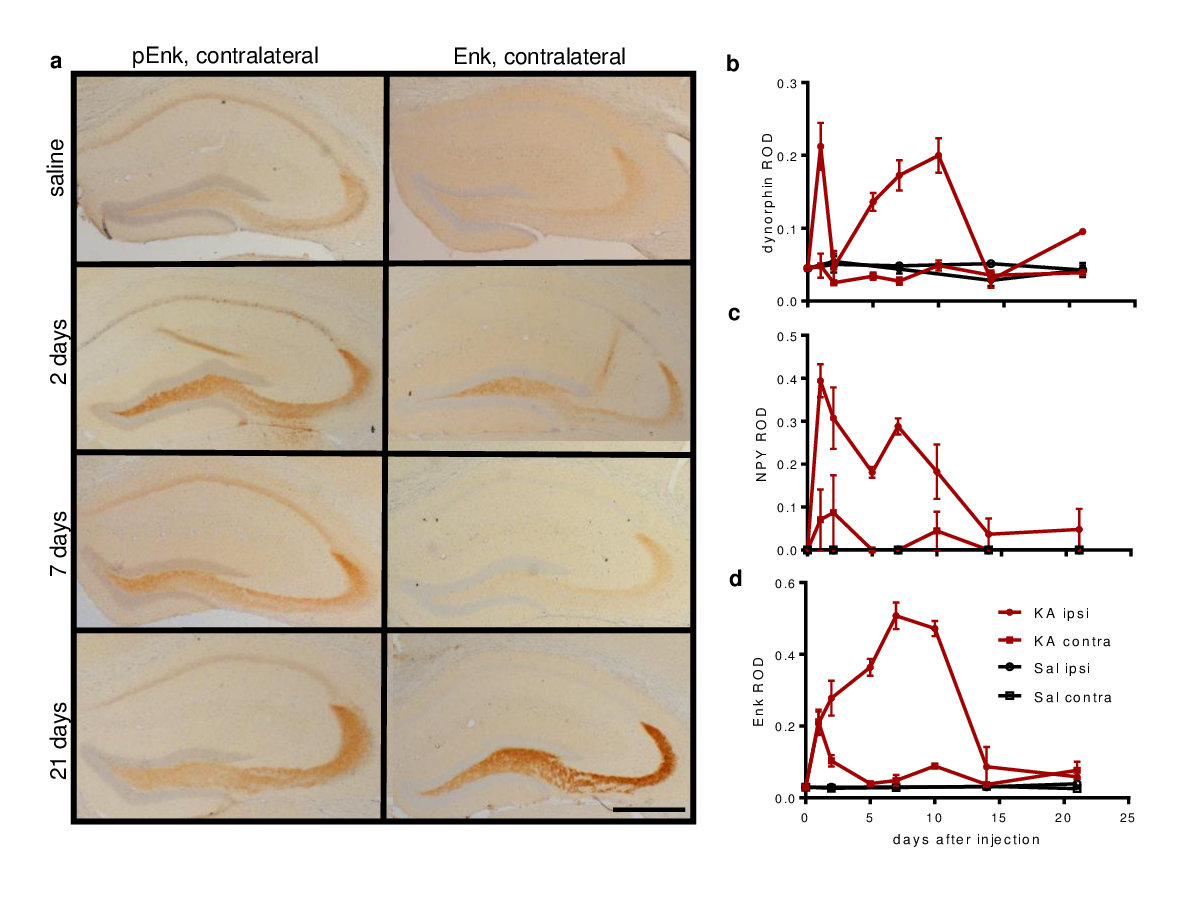

Supplement: FIGURE S1 — (Related to Figures 1–3). Enkephalin (Enk) protein- and mRNA-levels during epileptogenesis are depicted. Images in (A) represent contralateral hippocampi stained for pro-Enk (pEnk) or mature Enk (Enk) after saline or kainic acid (KA) injection at different time intervals. mRNA-levels in the dentate gyrus of WT mice for dynorphin (B), neuropeptide Y (NPY, C), and Enk (D) during epileptogenesis are depicted as relative optical densities measured from film autoradiographs obtained by radioactive in situ hybridization. Scale bar in (A) indicates 500 μm. [file Image_1.JPEG]

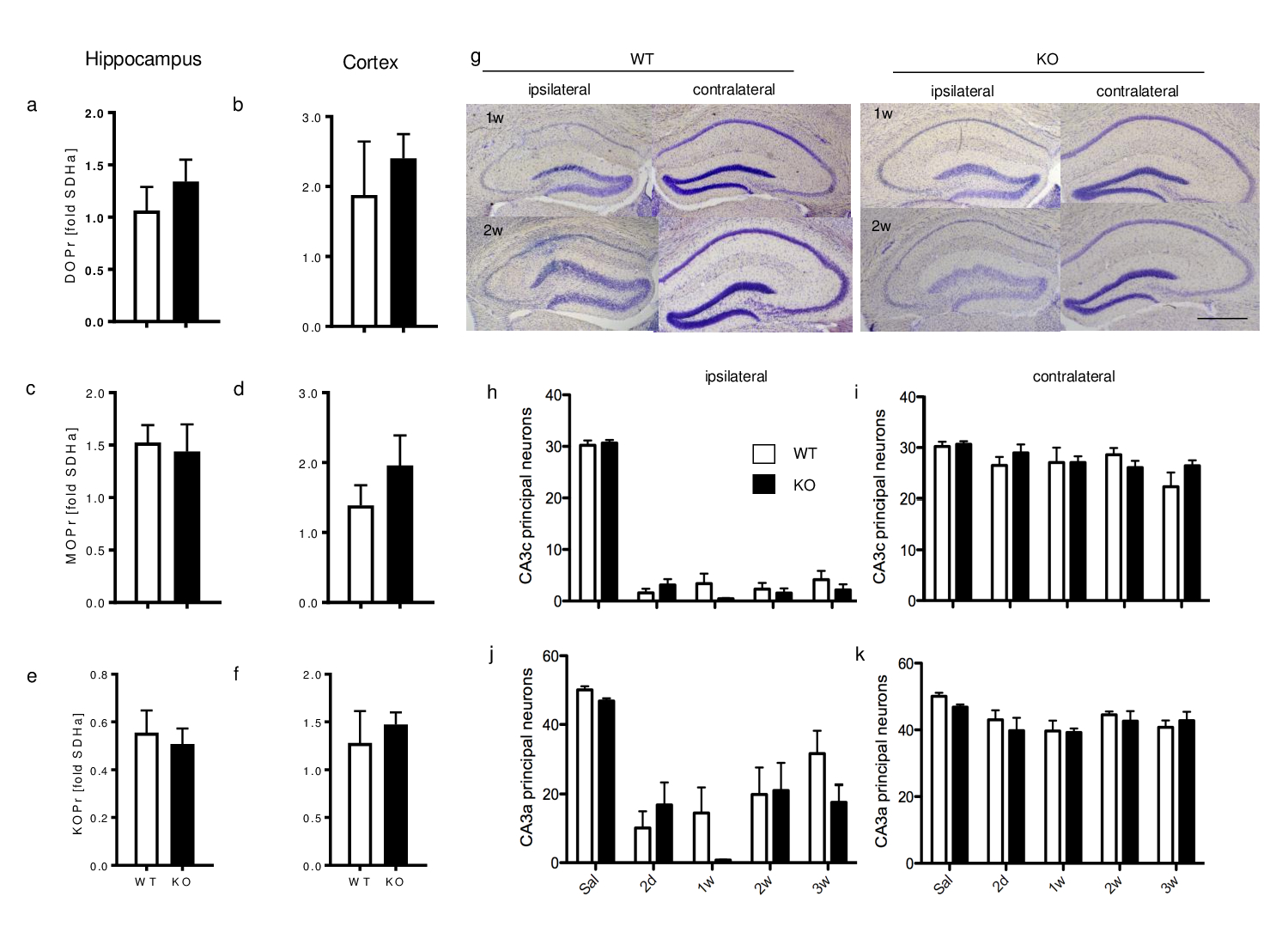

Supplement: FIGURE S2 — (Related to Figure 2). mRNA-levels of opioid receptors in WT and Enk-/- mice (KO) and additional data on cell numbers are depicted. mRNA levels were assessed for delta-, mu-, and kappa-opioid receptors (DOPr, MOPr, KOPr, respectively) in the hippocampus and cortex of young adult male mice (A–F). Nissl-stained hippocampal sections 1 week (1w) and 2 weeks (2w) after KA injection (G) show the onset of granule cell dispersion. Cell counts of area CA3 subfields are given in (H–K). Scale bar indicates 500 μm. (A–F) Cortex and hippocampi from naive adult WT and Enk-/- mice (N = 4) were analyzed for expression of mu, delta, and kappa opioid receptors (Taqman primer sets Mm01188089_m1; Mm01180757_m1; Mm01230885_m1; respectively; Thermo Fisher) by qPCR. Reactions were performed in a MicroAmp Fast Optical 96-Well Reaction Plate (Applied Biosystems) using the 7500 Fast Real-Time PCR System (Applied Biosystems). Samples were run in duplicates using 50 ng of total RNA equivalents (cDNA). Positive and negative controls were included in all experiments. Threshold cycle (CT) values were recorded as a measure of initial template concentration. Relative levels of RNA were calculated by the ΔΔCT method using SDHa as a reference standard gene. The fold-difference expression was calculated relative to a calibrator sample by 2-ΔΔCT. [file Image_2.JPEG]

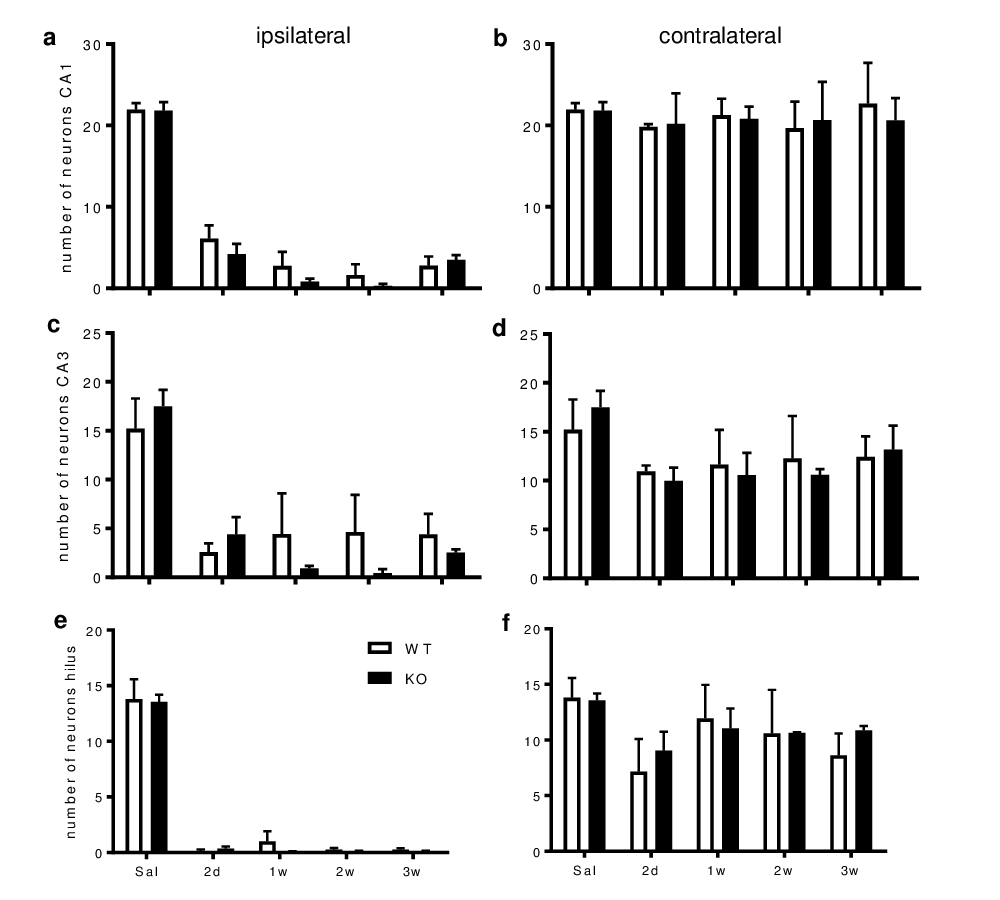

Supplement: FIGURE S3 — (Related to Figure 3). Numbers of somatostatin immuno-positive neurons were analyzed from WT and Enk-/- mice (KO) in the hippocampal subfields at different time-intervals after KA. Almost complete cell loss was observed ipsilaterally (A,C,E). Somatostatin positive neurons were mostly conserved contralaterally (B,D,F). No genotype specific differences in cell numbers were detected applying 2way ANOVA on distinct hippocampal areas. N per condition = 3–4. Ipsilaterally (left panels), time effects were significant in all hippocampal areas (F = 157.1; F = 17.6; F = 119 from top to bottom, P in all cases < 0.0001), but not genotype- or interaction-effects. Cell numbers represent area under the curve (AUC) across six hippocampal sections from 1.4 to 2.4 mm caudal to bregma. [file Image_3.JPEG]

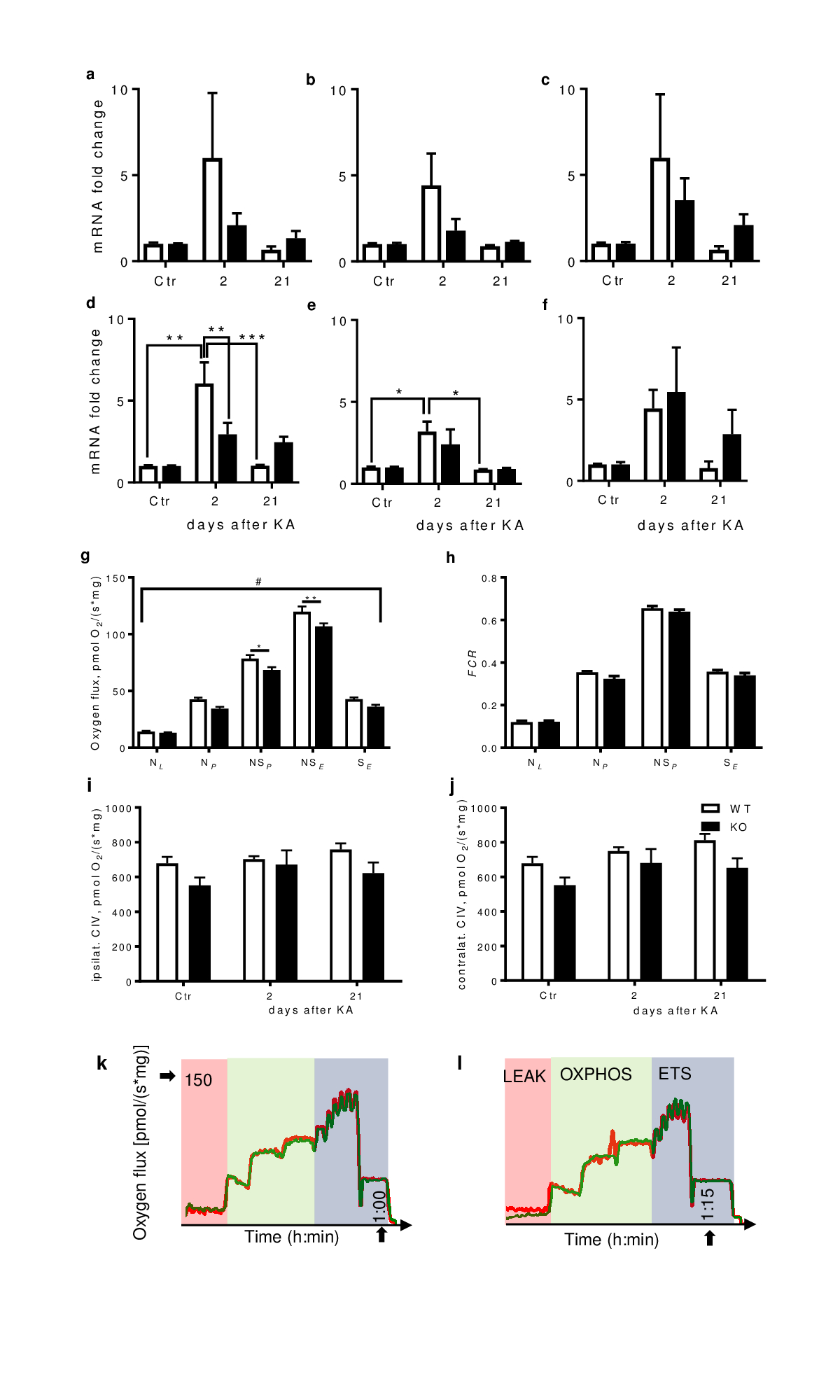

Supplement: FIGURE S4 — (Related to Figures 4, 5). mRNA levels of selected subunits of respiratory complexes of WT and enkephalin deficient mice (KO), normalized to beta-actin and basal levels of untreated animals are depicted: NDUFs3 (A,D), SDHb (B,E), and ATP6 (C,F). Data from (A–C) have been obtained from the hippocampus of the hemisphere of injection, data from (D–F) from the contralateral hippocampus. Absolute oxygen fluxes (G) of naïve WT and KO mice differed in a 2way repeated measures ANOVA [Finteraction(4,64) = 3.51, P = 0.012/Fstate(4,64) = 1073, P < 0.001, Fgenotype(1,16) = 7.52, P = 0.015], but not flux control ratios (FCRs, H). No changes were observed for Complex IV respiration across genotypes and time intervals after KA (I,J). Representative traces of respiration protocols for naïve controls are depicted for WT (K) and KO (L). NADH-linked respiration (NL and NP), OXPHOS capacity (NSP), ET capacity (NSE), succinate-linked respiration (SE). #P < 0.05, ∗P < 0.05, ∗∗P < 0.01, and ∗∗∗P < 0.001. [file Image_4.JPEG]
